# NeuroPID: a classifier of neuropeptide precursors

**DOI:** 10.1093/nar/gku363

**Published:** 2014-05-03

**Authors:** Solange Karsenty, Nadav Rappoport, Dan Ofer, Adva Zair, Michal Linial

**Affiliations:** 1 School of Computer Science and Engineering, The Hebrew University of Jerusalem, Jerusalem, Israel; 2 School of Computer Science, Hadassah Academic College, Jerusalem, Israel; 3 Department of Biological Chemistry, The Alexander Silberman Institute of Life Sciences, The Sudarsky Center for Computational Biology, The Hebrew University of Jerusalem, Jerusalem, Israel

## Abstract

Neuropeptides (NPs) are short secreted peptides produced in neurons. NPs act by activating signaling cascades governing broad functions such as metabolism, sensation and behavior throughout the animal kingdom. NPs are the products of multistep processing of longer proteins, the NP precursors (NPPs). We present NeuroPID (Neuropeptide Precursor Identifier), an online machine-learning tool that identifies metazoan NPPs. NeuroPID was trained on 1418 NPPs annotated as such by UniProtKB. A large number of sequence-based features were extracted for each sequence with the goal of capturing the biophysical and informational-statistical properties that distinguish NPPs from other proteins. Training several machine-learning models, including support vector machines and ensemble decision trees, led to high accuracy (89–94%) and precision (90–93%) in cross-validation tests. For inputs of thousands of unseen sequences, the tool provides a ranked list of high quality predictions based on the results of four machine-learning classifiers. The output reveals many uncharacterized NPPs and secreted cell modulators that are rich in potential cleavage sites. NeuroPID is a discovery and a prediction tool that can be used to identify NPPs from unannotated transcriptomes and mass spectrometry experiments. NeuroPID predicted sequences are attractive targets for investigating behavior, physiology and cell modulation. The NeuroPID web tool is available at http:// neuropid.cs.huji.ac.il.

## INTRODUCTION

The functions elicited by neuropeptides (NPs) cover most aspects of metazoan life including metabolism, growth and social behavior (1). For example, in mollusks and insects, mating behavior and reproduction are regulated by NPs. More generally, NPs function in energy consumption, food uptake, pain and sensation, temperature control, appetite, mating and social behavior. NPs act through binding their cognate receptor and activating a signaling cascade (2).

Most mature NPs are short peptides (5–30 aa) that are produced from a longer polypeptide, referred to as a NP precursor (NPP). Active NPs are the products of multiple cleavages of the precursor. Such cleavages mostly occur at basic residues (Arg and Lys) motifs that flank the NPs. While dibasic residues are the hallmark of the cleavage sites for endopeptidases (3), in some NPs such canonical cleavage sites are not detected.

Several bioinformatics approaches were developed to enhance the routine BLAST searching protocol by incorporating distinctive properties of NPs (e.g. (4)). However, at present, there is no systematic approach to identify NPPs in poorly annotated metazoan genomes. The organization of the NPP as a source of multiple NPs is conserved despite a weak similarity between NPPs along the evolutionary tree (5). Currently, NPPs are sporadically identified from sequencing projects of organisms and from the collections of large-scale proteomics and transcriptomics experiments. We developed a systematic approach for identifying candidate NPPs (6). Large-scale mass spectrometry (MS) proteomics identified thousands of spectra that derived from NPP genes. Additionally, a large number of bioactive modulatory peptides were identified (e.g. (7)). For peptides collected from human samples, only about 20% of the associated genes are currently annotated as NPPs. The expansion in MS proteomics and the depth of the transcriptome coverage suggest that NeuroPID is valuable as a discovery platform for bioactive modulators. NPPs and their associated NPs are attractive targets for drug development and pharmacological manipulations.

## OUTLINE—FEATURES TO PREDICTIONS

**Figure 1.**
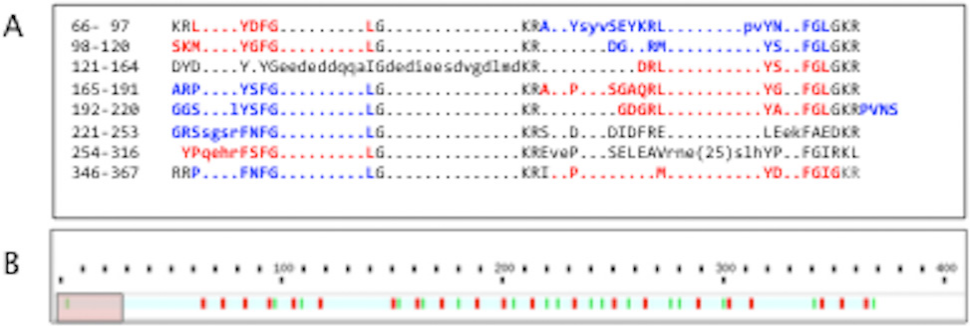
Allatostatin NPP from *Diploptera punctata* (Pacific beetle cockroach). **(A)** The sequence (P12764, ALLS_DIPPU, 370 aa) has 7 weak ‘repeats’. The repeated segments account for Allatostatin-1 to Allatostatin-13. The NPs are colored red and blue interchangeably. **(B)** NeuroPID visualization of P12764. The rectangle at the N′-terminal indicates the Signal Peptide (SP). Arg (R) or Lys (K) and dibasic residues are colored green and red, respectively.

Figure 1 shows a prototype of an NPP that belongs to the Allatostatin family as an illustration for the challenges in identifying such genes at a genomic scale. The Allatostatins are produced in many insects to control food intake (8). The sequence of the Pacific beetle cockroach (Figure 1) contains 13 identified NPs. While each peptide has a unique sequence, all share the Tyr/Phe-Xaa-Phe-Gly Leu/Ile-NH2 consensus. The weak sequence similarity among the NPs emphasizes the challenge in identifying NPPs from unannotated genomes.

The classifier underlying NeuroPID was derived by a supervised machine-learning (ML) methodology on a set of true and false instances obtained from 1418 manually annotated NPPs from the UniProtKB database. Each sequence is converted into a vector of primary sequence-derived features (∼560 features). The features cover the amino acid composition, bigrams frequencies (400 features) and additional 140 biophysical and statistical characteristics from the sequence (6). The performance of the NeuroPID was assessed using different predictive models. The positive and negative sets included ∼1000 sequences each. We randomly split the sequences and carried out 6-fold cross-validation, with a fraction of the data (i.e. 10–40%) serving as a disjoint set for the iterations during the training phase. The training was performed in view of several independent negative sets (e.g. nuclear proteins, secreted membranous proteins). The results of the tests for each of the validation set was summed and averaged to estimate the MCC (Matthews correlation coefficient) and AUC (area under receiver operating characteristic curve). The statistical definitions of accuracy, precision and recall used are:

- ${\rm Accuracy} = \frac{{{\rm TP} + {\rm TN}}}{{{\rm TP} + {\rm FP} + {\rm TN} + {\rm FN}}}$${\rm Accuracy} = \frac{{{\rm TP} + {\rm TN}}}{{{\rm TP} + {\rm FP} + {\rm TN} + {\rm FN}}}$
- ${\rm Precision} = \frac{{{\rm TP}}}{{TP + FP}}$${\rm Precision} = \frac{{{\rm TP}}}{{TP + FP}}$
- ${\rm Recall}({\rm sensitive}) = \frac{{{\rm TP}}}{{{\rm TP} + {\rm FN}}}$${\rm Recall}({\rm sensitive}) = \frac{{{\rm TP}}}{{{\rm TP} + {\rm FN}}}$

**Table 1.** Performance of ML classification models implemented in NeuroPred

ML model	Accuracy	Precision	Recall	MCC	AUC
Random Forest	0.928	0.938	0.915	0.857	0.928
Gradient Boosting	0.93	0.928	0.929	0.859	0.93
Linear SVM	0.881	0.87	0.894	0.763	0.882

where TP, TN, FP and FN are the true positives, true negative, false positive and false negative, respectively. Table 1 summarizes representative ML models with AUCs ranging from 0.89 to 0.93. Testing many sets of sequences that were not included in the ML training (a blind test) resulted in high quality predictions. For example, testing 3000 Amphibian sequences, resulted in 62 positive predictions, 34 of them were marked as high quality predictions. Manual inspection revealed 3/34 as false positives while the rest of the predictions include classical NPs, secreted peptide from skin and modulators of the immune system. This result shows the high success rate of NeuroPID, and the relative enrichment of NPs in Amphibian proteomes.

An additional test included human peptides from resources for NPs and bioactive peptides. There are 94 protein sequences that account for the 270 identified peptides from human samples (7). Recall that only 18/94 sequences are currently annotated ‘neuropeptide’ by UniProtKB. An input of all 94 human protein sequences to NeuroPID positively predicted 85% (80/94) of these sequences, with 67.5% as high quality predictions. Using additional annotation resources (mostly Gene Ontology - GO) extended the number of sequences that are related to NPs to 39 sequences. NeuroPID identified all the 39 sequences as positive predictions (with 72% as ‘top quality predictions’). Therefore, we estimate the discovery rate of NeuroPID to range between 68% and 85%. A similar test on all ∼400 human bioactive peptides from SwePep (9) showed that NeuroPID recovers 72% of the relevant proteins as positive predictions.

## SEQUENCED GENOMES—A SOURCE OF NPP CANDIDATES

At present, there are tens of metazoan genomes that are fully or partially sequenced. An attractive application for NeuroPID is in screening unannotated transcriptomes. There are over 25 complete insects’ genomes including 12 Drosophilae (10), social insects (e.g. honey bee), vector of pathogens (e.g. Anopheles) and more. The input for NeuroPID can be extracted directly from genomic annotation projects (e.g. Hymenoptera database (11)), and from the major protein resources such as the UniProtKB (12) and NCBI (National Center for Biotechnology Information) protein databases (13). Complementary resources include cDNA libraries, ESTs (expressed sequence tags) and RNA-Seq reads. Another rich source for NeuroPID input are sequences from MS experiments (14).

## USER PERSPECTIVE AND WEB INTERFACE

NeuroPID is described for a typical user seeking to identify NPPs from a large collection of unknown sequences.

### Input's page

This page includes text entry and file uploading options for FASTA format protein sequences. The system accepts thousands of sequences. There is no limit on the number of input sequences, and tens of thousands of sequences can be handled. An option to activate the SignalP 4.0 program for additional filtering (15) is provided. SignalP computationally predicts the presence of a signal peptide (SP) at the N′-terminal of each sequence. The SP filter may increase the run time (up to several minutes for more than 10 000 sequences). Note that the various protein resources (Pfam, SwissProt, NCBI-proteins) are not necessarily consistent in defining SP.

### Results’ page

**Figure 2.**
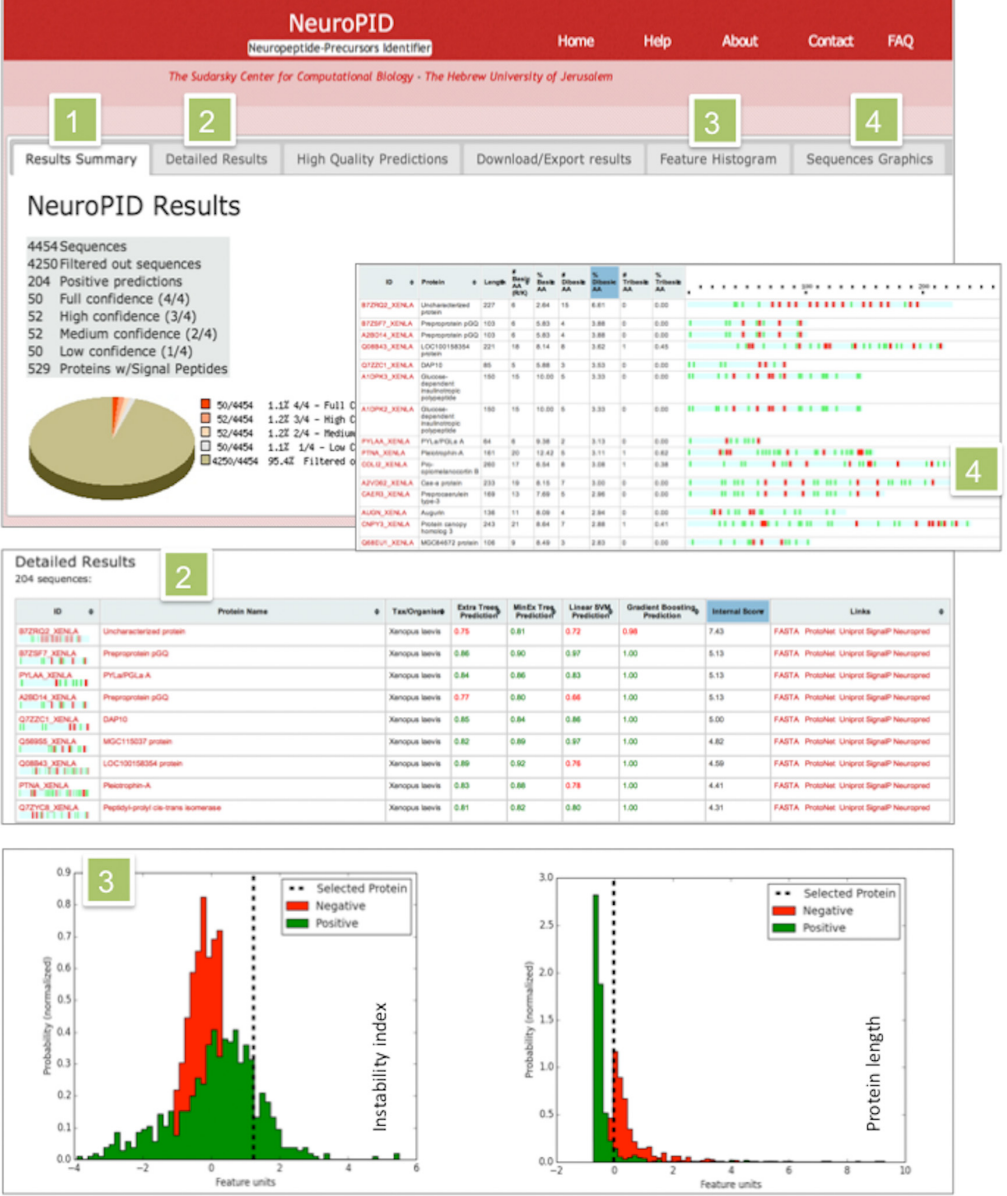
Screenshots of NeuroPID prediction page **(1)** A summary table for an input of 4454 proteins from *Xenopus laevis*. A pie chart displays the distribution of prediction according to the agreement of the classifiers. **(2)** A detailed table shows the confidence for each prediction methods. The red and green fonts indicate negative and positive predictions, respectively. We consider a prediction to be a positive prediction according to predictor-specific threshold. The table is ranked by the Internal Score (IS). The sequence is linked to its FASTA sequence, to knowledge-based resources (ProtoNet, UniProtKB) and analysis tools (SignalP, NeuroPred). **(3)** Results from the feature histogram. The position of a selected protein (dash line) in view of the distribution of the positive and negative sets is shown. The distributions are shown in green and red colors to indicate the positive and negative instances, respectively. The data were normalized by the standard deviations of the distributions (denoted Feature Units, *X*-axis). **(4)** Sequences graphics according to protein's length and positions of basic residues. The red vertical line on the sequence shows the location of dibasic and tribasic residues. The presence of a single basic residue is colored green.

This page includes a *Summary Table* with a schematic partition of the positive and negative predictions (Figure 2, marked ‘1’). The system removes sequences with non-legitimate residues (i.e. B, X, U or Z). In the example in Figure 2, among the 4454 sequences, only 529 proteins have predicted to have SP, and positive predictions comprise only 4.6% of the initial input.

The *Detailed Results* are presented as a table that specifies the probabilistic scores of each ML prediction model (Figure 2, marked ‘2’) and an indication for the positive predictions (i.e. the protein is candidate for NPP, green font). The threshold on the probabilistic score is >0.8 for Linear SVM (support vector machine), the Extra trees and the Minimal tree predictions, and >0.99 for the Gradient Boosting classifier. The user can sort each column and thus rank the list by the preferred method of prediction, or by the Internal Score (IS), which provides an additional high quality filter (see ‘Navigating among NeuroPID predictions’).

Each sequence is linked to a further downstream analysis, including ProtoNet classification for protein clustering (16), SignalP which provides a confidence score for having a classical SP in the sequence (15), NeuroPred which predicts the likelihood of NPP processing into NPs based on the predicted cleavage products (17). In addition, the sequence is linked to the protein sequence's page from UniProtKB. The FASTA sequence can also be retrieved for further analysis (Figure 2, marked ‘2’).

A tab for *High Quality Predictions* shows a subset of the positive predictions above a pre-selected IS threshold. This list is considered a reliable set for NPPs and neuromodulators. Note that the IS threshold used for the ‘top quality predictions’ relies strongly on the presence and distribution of di- and tribasic motifs along the sequence. Evidently, NPPs that are not processed by the canonical proteolysis pattern will not be included in such list. Therefore, browsing among all NeuroPID positive predictions is advisable.

Batch downloading of the data is available with the *Download* / *Forward* options. The list of all positive predictions can be directly forwarded to NeuroPred. In addition, the detailed results can be downloaded for further analysis by inference tools such as PANDORA (18).

The *Feature Histogram* tab provides a visual analysis tool for a specific feature and a selected protein. The features listed contributed significantly to the NeuroPID classifiers performance. In Figure 2 (marked ‘3’), the position of a selected protein (dash line) in view of the distributions of the features of ‘protein length’ and ‘instability index’ over the positive (green) and negative (red) sets is shown.

The *Sequence Graphics* presentation tab provides visualization for each sequence according to its length and the appearance of basic residues (R and K) along the sequence. The quantitative value for the number of the motifs and their density are indicated (Figure 2 marked ‘4’). Note that only sequences processed by dibasic proteolysis (The ‘Known Motif’ model) (16) will benefit from measuring the high density of dibasic and tribasic motifs. An example of Allatostatin is shown in Figure 1B. The Sequence Graphics visualization is not restricted to sequences that lack the potential dibasic cleavage sites.

### Help page

NeuroPID helps new users by providing answers to Frequently Asked Questions. In addition, it provides a brief explanation on the different visualization options.

The major protein sequences resources are listed. The UniProtKB search engine helps users in retrieving sequences according to a list of IDs or accession numbers (13). In addition, NeuroPID lists supporting tools that can be used to test the input sequences for other functions such as the short toxin-like modulatory proteins (19), PANDORA that provides annotation-driven analysis and visualization for sets of sequences (18) and more.

## NAVIGATING AMONG NEUROPID PREDICTIONS

We provide a short guideline based on testing thousands of sequences from many different organisms. Applying additional filter to the positively predicted sequences is a good practice for limiting the number of false positive predictions:

- *Removal of sequences that lack SPs.* All NPPs are secreted proteins carrying a SP at the N′-terminals. We encourage the user to apply the filter of SP predictor on the input set. For example, an input of 750 size-limited proteins (<300 aa) from the common frog *Rana temporaria* resulted in 88 positive predictions, but only 2 positive predictions are reported when the SP filter is applied.
- *Confidence of predictions.* The positive predictions are proteins that are predicted by at least one of the four prediction models (Random Forest-decision tree ensemble and SVM-based models). While the Extra Tree model uses all 560 features for the prediction phase, the minEx Tree relies only on a small set of the most discriminative features. Consequently, the results from the two classification models are highly correlated. The user can select results that are more consistent between the four ML prediction models for increasing reliability.
- *High quality predictions have a high density of dibasic residues.* In addition to the presence of SP, the characteristics of the dibasic cleavage site are used as additional filters for restricting the results to the high quality set. Dibasic and tribasic motifs play a crucial role in the cleavage and processing of NPPs (Figure 1). We designed an IS that captures the density of dibasic and tribasic residues and calculate the percentage of such motifs in the effective length of the protein (after removal of s typical SP length of 25 aa). The user is encouraged to use the *high quality prediction* filtered list ranked by the IS values. From an input of 4454 sequences from *Xenopus laevis* (<300 aa), NeuroPID reported on 204 positive sequences but only 69 sequences as high quality NPP candidates.

## FUNCTIONAL INFERENCE

Analyzing the *high quality predictions* for functional enrichment of keywords reveals enrichment of several keywords including proteins of the innate immune response such as antimicrobial peptides. Additional proteins include growth factors, hormonal peptides, extracellular receptor fragments, proteases and insulin-like proteins. All of these proteins are short, secreted cellular modulators.

Most sequences from newly sequences genomes and from MS proteomics resources are uncharacterized. The top predicted list from NeuroPID should be further analyzed using independent tools. We allow the predicted sequences to be further analyzed by the ProtoNet hierarchical tree (16). ProtoNet clusters typically provide a rich set of annotations associated with the members of the relevant cluster. We provide a forwarding option to NeuroPred that assess the probability of a sequence to be processed for bioactive peptide products (17).

## UPDATE AND FUTURE DEVELOPMENT

The NeuroPID and its pipeline of data transformation and feature generation are implemented using Python and the scikit-learn toolkit (20). Web server developers that are interested in interfacing directly with NeuroPID should contact the authors. The NeuroPID code, resources of sequences and additional information are available upon request. Source code for the underlying predictive framework used herein and in the original article, is freely available online: http://www.protonet.cs.huji.ac.il/neuropid.

NeuroPID will be extended in few directions: (i) Locally adding downstream analysis tools. (ii) Allowing inputs of peptides that originate from proteomics MS experiments. We will use the implementation scheme of PANDORA (18). (iii) Archiving a catalog of NeuroPID best predictions as candidates for future experimental validation.

We expect that NeuroPID will be useful in leveraging the NPPs annotation and the discovery of overlooked modulators. NeuroPID has the potential for generating biotechnological leads for the metabolic status, social behavior and cells' communication.
